# Short-term consequences of *Helicobacter pylori* treatment in patients with oral lichen planus: A prospective study

**DOI:** 10.34172/japid.2023.004

**Published:** 2022-10-09

**Authors:** Farshad Javadzadeh, Masoud Shirmohamadi, Maryam Hosseinpour Sarmadi, Morteza Ghojazadeh, Sepideh Bohlouli, Amir Ghorbanihaghjo, Solmaz Pourzare

**Affiliations:** ^1^Department of Oral Medicine, Faculty of Dentistry, Tabriz University of Medical Sciences, Tabriz, Iran; ^2^Liver and Gastrointestinal Diseases Research Center, Tabriz University of Medical Sciences, Tabriz, Iran; ^3^Research Center for Evidence-Based Medicine, Iranian EBM Centre: A Joanna Briggs Institute Affiliated Group, Tabriz University of Medical Sciences, Tabriz, Iran; ^4^Biochemistry and Clinical Laboratories Department, Biomedical Research Institute, Tabriz University of Medical Sciences, Tabriz, Iran

**Keywords:** Helicobacter pylori, Oral lichen planus, Visual analog scale

## Abstract

**Background.:**

Oral lichen planus (OLP) is a multifactorial chronic inflammatory condition with unknown etiology. This condition has been associated with *Helicobacter pylori*. This study aimed to investigate the relationship between the treatment of *H. pylori* infection and improvements in OLP lesions.

**Methods.:**

In this cohort study, 42 patients with erosive or ulcerative OLP lesions were evaluated in terms of *H. pylori* infection using the *H. pylori* stool antigen (HpSA) test. The patients were divided into three groups. The first group consisted of 12 *H. pylori*-negative patients. The second group consisted of 21 *H. pylori*-positive patients receiving antibacterial treatment. The third group included nine *H. pylori*-positive patients not willing to receive treatment. All the three groups underwent the usual OLP treatment. Patients in the second and third groups were re-evaluated by the HpSA test after two months. The efficacy indexes and visual analog scale were used to evaluate clinical improvements.

**Results.:**

The efficiency index and pain scores were affected by the intervention (*P*<0.001). The logistic regression analysis showed that the severity index before treatment was significantly effective (OR=0.745 (95% CI: 0.602‒0.923; *P*=0.007). No statistical significance for factors affecting other variables (*P*>0.05) was obtained.

**Conclusion.:**

Pain intensity was higher in patients with *H. pylori* than in those without *H. pylori* before treatment. Also, in patients with *H. pylori*, the treatment affects the complete recovery rate.

## Introduction

 Oral lichen planus (OLP) affects approximately 2% of the population. It has several overlapping morphological forms, including reticular, erosive, popular, vesiculobullous, and atrophic/erythematous.^1^ Erosive oral lichen planus (EOLP) is characterized by erythema and erosion that predominantly affect the buccal mucosae. Nonetheless, 25%–30% of patients might have gingival involvement with diffuse pain, erythema, and erosions, resulting in desquamative gingivitis.

 Oral lichenoid reactions are a group of lesions with different etiologic factors and clinical and histological manifestations, including OLP, lichenoid contact reaction, lichenoid drug rash, lichenoid reaction, and graft-versus-host disease (GvHD). OLP is a chronic inflammatory condition with still unknown etiology that affects the oral mucosa and is considered an autoimmune disease of T cells.^2^ Despite contradictory results, some studies suggest OLP as a premalignant lesion with the potential to develop squamous cell carcinoma. Different studies have reported a 3.5%–4% malignancy rate.^3,4^

*Helicobacter pylori* is a gram-negative, flagellate, and helical bacterial species in the stomach, associated with chronic gastritis, gastric ulcer, and gastric adenocarcinoma. *H. pylori* infection is one of the world’s most common bacterial infections. It is believed that almost half of the world’s population is infected with this microbe. Also, 80% of the infections are reported in developing countries, with 20%–50% in developed countries.^5^ Although the exact transmission route is still debated, some evidence suggests human-to-human contact as the most likely route.

 In addition to the stomach, this bacterium is also found in dental plaque and feces. The role of dental plaque as a reservoir of *H. pylori* and a possible source of infection or re-infection of the gastric mucosa has been discussed for a long time.^6^ Since OLP is a lesion with multifactorial etiology, *H. pylori* infection has been considered an important factor in its pathogenesis. Moreover, several studies have reported a positive relationship between OLP and *H. pylori*, reporting a higher prevalence among females than males.^5,6^

 Although most studies have confirmed the association between *H. pylori *and OLP lesions, currently, no study is available on the effect of treating *H. pylori *infection on improving OLP lesions. Therefore, this study aimed to evaluate the frequency of *H. pylori *in patients with erosive and ulcerative OLP. Also, we investigated the relationship between short-term therapeutic effects of *H. pylori *infection on the improvement of OLP lesions based on clinical criteria, including efficacy index (EI) and visual analog scale (VAS).

## Methods

###  Study groups

 We followed the guidelines of the STROBE checklist. The statistical population included all patients with erosive or ulcerative OLP lesions. Fifty-one patients with OLP referred to the Oral and Maxillofacial Department of Tabriz Dental School, Iran, participated in the study. Nine patients were excluded from the study due to poor cooperation in receiving *H. pylori* drugs and incomplete treatment or unwillingness to participate in a second trial to evaluate the improvement in *H. pylori*. Finally, 42 patients (female: 27 vs. male: 15) completed the study. Informed consent was obtained from all the patients prior to the study. The Ethics Committee of Tabriz University of Medical Sciences approved the study (code: IR.TBZMED.REC.1400.063).

 The clinical signs and biopsy were confirmed by an oral and maxillofacial medicine specialist. A histopathological examination was performed by a pathologist. All the patients with erosive and ulcerative OLP were evaluated and divided into three groups based on the *H. pylori* stool antigen (HpSA) test results. These patients signed informed consent forms to receive treatment. The first group (n = 12) included patients with OLP lesions without infection. The second group (n = 21) included patients with OLP lesions, gastrointestinal infection with *H. pylori,* and those receiving four-drug treatment in addition to routine OLP treatment. The third group (n = 9) included patients with an OLP lesion who did not want to receive medical treatment despite the positive test result.

###  The inclusion criteria

 The inclusion criteria were as follows: patients with OLP lesions with symptomatic malignant potential, including erosive and ulcerative OLP, erosive and ulcerative lichenoid contact reaction, and erosive and ulcerative drug-induced lichenoid reactions following clinical and histopathological criteria.^7^

###  The exclusion criteria

 The exclusion criteria were as follows: use of antibiotics in the past month, proton pump inhibitors (PPI) in the last two weeks, and any H2 receptor blockers in the last 48 hours; patient’s unwillingness to participate in the study.

###  Treatment 

 The three groups underwent routine OLP treatment, including topical steroids combined with a mouthwash containing Al-Mg and 15 betamethasone injections for three weeks, followed by tapering over the next six weeks. All the patients were evaluated on three occasions: at the first visit, four weeks later, and nine weeks after starting the treatment. The treatment in the second group included a four-drug sequential treatment plan consisting of omeprazole capsules (40 mg) before breakfast, amoxicillin capsules (500 mg) in the first five days twice a day (two capsules each time), two clarithromycin (500 mg) tablets in the second five days (once a day), and two tinidazole (500 mg) tablets in the second five days (once a day).

###  Clinical and laboratory assessments

 A gastroenterologist performed the HpSA test again to check the status of the gastrointestinal infection. It should be noted that in a small number of patients requiring systemic corticosteroids, *H. pylori* treatment did not interfere with systemic corticosteroids. The clinical criteria, including VAS, SI, and EI, were used to evaluate the clinical improvement before and after treating gastrointestinal *H. pylori* and conventional OLP treatment. The pain intensity was determined before and after treatment by the visual analog scale (VAS) method. A 10-cm line was drawn on paper. The patients were requested to mark pain intensity on the line without being stimulated. The pain score ranged from zero (painless) to 10 (very severe pain), and the numerical value was recorded as VAS.^8^

###  EI calculation method

 V_0_ = Wound size on the first day of referral

 V_60_ = Wound size two months after the first visit

 EI = [(V_0_ – V_60_) / V_0_] × 100%

1. Heal: EI = 100%
2. Marked Improvement: 75% < EI < 100%
3. Moderate improvement: 25% < EI < 75%
4. Mild improvement: 0% < EI < 25%
5. No improvement EI = 0%.^9^

###  Statistical analysis

 The results of descriptive statistics for qualitative variables were reported in frequencies and percentages. Chi-squared and Fisher’s exact tests were used to compare the ratios between the two dependent groups. Univariate logistic regression was used to investigate the effect of *H. pylori* on the dependent variable of treatment. All the analyses were performed using SPSS 26. A *P* < 0.05 was considered statistically significant.

## Results

 It was observed that 71% of OLP patients had *H. pylori *infection. Among the *H. pylori*-positive subjects, 56% had ulcerative OLP lesions, and 44% had erosive lesions. Among the *H. pylori*-negative patients, 75% had ulcerative lesions, and 25% had erosive lesions. Also, 70% of *H. pylori*-positive OLP patients received routine OLP treatment, while 30% were reluctant to undergo treatment. The oral lesions in 70% of *H. pylori*-positive patients completely recovered, and the lesions improved in 83.3% of patients with no evidence of gastrointestinal infection. Based on the VAS method, the pain severity in *H. pylori-*positive patients was higher than in *H. pylori-*negative ones. After routine treatment of OLP, while the pain intensity improved in all *H. pylori*-negative patients, it improved in 86% of *H. pylori*-positive patients (Table 1).

**Table 1:** Frequency of studied groups and clinical parameters

**Variable**		**Frequency N (%)**
Oral lichen planus type	Ulcerative	26 (61.9)
	Erosive	16 (38.1)
*H. pylori* before treatment	Negative	12 (28.6)
	Positive	30 (71.4)
*H. pylori* after treatment	Negative	33 (78.6)
	Positive	9 (21.4)
EI with positive *H. pylor*i before treatment	EI ≤ 25	4 (13.3)
	25 < EI ≤ 75	5 (16.7)
	EI ≥ 75	21 (70)
EI with negative *H. pylori* before treatment	EI ≤ 25	0
	25 < EI ≤ 75	2(16.7)
	EI ≥ 75	10(83.3)
Pain score with positive *H. pylor*i before treatment (VAS)	Mild	10(23.8)
	Moderate	16(38)
	Severe	4(9.5)
Pain score with negative *H. pylori* before treatment (VAS)	Mild	7(16.7)
	Moderate	4(9.5)
	Severe	1(2.4)
Pain score with positive *H. pylori* after treatment (VAS)	Mild	5(55.6)
	Moderate	4(44.4)
	Severe	0
Pain score with negative *H. pylori* after treatment (VAS)	Mild	21(100)
	Moderate	0
	Severe	0

N total: The number of patients EI: Efficiency index, VAS: visual analog scale

 According to the results of EI, OLP lesions completely recovered in patients with *H. pylori *infection, who received treatment, and *H. pylori-*negative patients compared to patients who did not receive treatment (*P* < 0.05).

 The recovery rate was higher (85.7%) among those who received treatment than those who did not receive full treatment (22.2%). The Chi-square test showed a statistically significant association between *H. pylori* after treatment and EI. (Table 2)

**Table 2:** Comparison of clinical parameters before and after treatment among studied groups

**Clinical parameters**		***H. pylori***** test**		***P*** ** value**
		**Positive **	**Negative **	
Efficiency index before *H. pylori *treatment	Ineligible improvement (%)	9 (30)	2 (16.7)	0.375*
	Healing and marked improvement (%)	21 (70)	10 (83.3)	
Efficiency index after *H. pylori *treatment	Ineligible improvement (%)	7 (77.8)	3 (14.3)	0.002*
	Healing and marked improvement (%)	2 (22.2)	18 (85.7)	
Pain score after *H. pylori *treatment (VAS)	Ineligible improvement (%)	4 (44.4)	0 (0.0)	0.005**
	Healing and marked improvement (%)	5 (55.6)	21 (100)	

VAS: visual analog scale. * Significant at 0.05 level by chi-square test. ** Significant at 0.05 level by Fisher’s exact test.

 Also, complete recovery was higher (100%) among those who received treatment than those who did not receive full treatment (55.6%). Fisher’s exact test showed a statistically significant association between *H. pylori *and VAS after treatment.

 Logistic regression analysis showed that the odds ratio (OR) in post-treatment *H. pylori*-positive subjects was 32.25 compared to post-treatment H*. pylori-*negative subjects (95% confidence interval: 3.9‒285.45). Since OR was > 1, it indicated that the chance of complete recovery in patients who received treatment was higher than in those who did not. The association was statistically significant (*P* < 0.05)

## Discussion

 OLP is a multifactorial disease with an unknown etiology. Several studies mentioned inflammation due to allergy to dental materials or reaction of infectious agents as the pathogenesis.^10^ Here, 71% of patients with OLP were *H. pylori*-positive and reported a higher pain intensity than *H. pylori-*negative patients. After treatment of OLP lesions, although the pain intensity improved in all patients without *H. pylori*, it improved in 86% of *H. pylori*-positive patients. According to the EI index, the complete recovery of OLP lesions showed a significant relationship with treatment in patients with *H. pylori* infection compared to those who did not receive treatment.

 Kazanowska-Dygdała et al^6^ reported a positive relationship between OLP lesions and *H. pylori*. Also, Khan et al^5^ showed a direct relationship between OLP and *H. pylori*, which was more pronounced in females than males. In another study by Viganò et al,^11^ *H. pylori*, OLP, and leukoplakia were positively correlated, consistent with the present study.

 However, Shimoyama et al,^12^ Taghavi Zenouz et al,^13^ and Hulimavu et al^14^ reported no association between OLP and *H. pylori*. The difference between the results of the present study and the studies mentioned above can be attributed to the relatively unknown etiology of OLP, despite its high prevalence. It seems that racial, geographic, and environmental factors are effective in the occurrence of the disease. Furthermore, the statistical population in the present study was different from the studies mentioned above. Also, some confounding factors, including age, sex, type of OLP (ulcerative and non-ulcerative), and type of *H. pylori *test, might affect have affected the results. Here, the HpSA test was performed by the ELISA method, which requires minimum cooperation from the patient. Also, stool samples can be stored for up to three days at 2‒8 °C and even more at -20 °C without any changes in the test results. However, HpSA testing is associated with limitations: the effect of fecal consistency on the test results and the non-uniform amount of *H. pylori *in all parts of the feces.

 Since this cohort study was conducted in a center for 15 months, the results should be interpreted with caution due to the different numbers of participants in each group. Therefore, studies with a longer duration, multi-center design, and larger statistical populations are recommended to confirm our results.

 In this study, the *H. pylori* fecal antigen test was used to investigate gastrointestinal infection. The test is more recent and offers high sensitivity and specificity.

## Conclusion

 In this study, pain intensity was higher in patients with *H. pylori *than in those without *H. pylori* before treatment. Also, in patients with *H. pylori*, treatment affected the complete recovery rate. Therefore, we witnessed that *H. pylori* affected pain intensity based on VAS, and the treatment of *H. pylori* affected EI or complete healing.

## Acknowledgments

 The authors would like to thank the patients for assisting in the study.

## Availability of Data

 The data that support our results can be accessed from the corresponding author. However, the data are unavailable to the public, as they contain private information about research volunteers.

## Competing Interests

 The authors declare that they have no competing interests.

## Ethical Approval

 Ethical approval number: 1400/063 IR.TBZMED.REC.

## Funding

 This work was funded by the Drug Applied Research Center of Tabriz University of Medical Sciences, Tabriz, Iran.
